# Collaborative Assessment and Health Risk of Heavy Metals in Soils and Tea Leaves in the Southwest Region of China

**DOI:** 10.3390/ijerph181910151

**Published:** 2021-09-27

**Authors:** Juan Liu, Weihong Lu, Naiming Zhang, Dan Su, Ladu Zeer, Hongdie Du, Kelin Hu

**Affiliations:** 1 College of Plant Protection, Yunnan Agricultural University, Kunming 650201, China; 15587214232@163.com (J.L.); luweihong_002@163.com (W.L.); 2 Yunnan Soil Fertility and Pollution Restoration Laboratory, Yunnan Agricultural University, Kunming 650201, China; Sd572018796@163.com (D.S.); ZELD196725@163.com (L.Z.); DuHongDieDHD@163.com (H.D.); 3 College of Resource and Environment Science, Yunnan Agricultural University, Kunming 650201, China; 4 College of Resource and Environment Science, China Agricultural University, Beijing 100094, China; hukel@cau.edu.cn

**Keywords:** tea leaves, heavy metal, health risk, tea plantation soil, impact index of comprehensive quality

## Abstract

The collaborative assessment and health risk evaluation of heavy metals (HMs) enrichment in soils and tea leaves are crucial to guarantee consumer safety. However, in high soil HM geochemical background areas superimposed by human activities, the health risk associated with HMs in soil–tea systems is not clear. This study assessed the HMs concentration (i.e., chromium (Cr), cadmium (Cd), arsenic (As), and lead (Pb)) in tea leaves and their relationship with soil amounts in the southwest region of China to evaluate the associated health risk in adults. The results revealed that the average soil concentration of Cr was the highest (79.06 mg kg^−1^), followed by Pb (29.27 mg kg^−1^), As (14.87 mg kg^−1^), and Cd (0.18 mg kg^−1^). Approximately 0.71, 4.99, 7.36, and 10.21% of soil samples exceeded the threshold values (NY/T 853-2004) for Pb, Cr, As, and Cd, respectively. Furthermore, the average concentration of Pb, As, and Cd in tea leaves was below the corresponding residue limits, but Cr was above the allowed limits. Correlation analysis revealed that the Pb, Cr, As, and Cd amounts in tea leaves were positively correlated to their soil amounts (*p* < 0.01) with an R^2^ of 0.203 **, 0.074 **, 0.036 **, and 0.090 **, respectively. Additionally, approximately 40.38% of the samples were found to be contaminated. Furthermore, spatial distribution statistical analysis revealed that Lancang was moderately contaminated, while Yingjiang, Zhenkang, Yongde, Zhenyuan, Lüchun, Jingdong, Ximeng, and Menglian were slightly contaminated areas. The target hazard quotients (THQ; health risk assessment) of Pb, Cr, As, and Cd and the hazard index (*HI*) of all the counties were below unity, suggesting unlikely health risks from tea consumption.

**Core ideas:**

- China has the largest tea plantation area and production in the world.
- Tea is unlikely to be a health risk to tea consumers.
- Heavy metals (HMs) enrichment in tea leaves is strongly linked to soil HMs amounts.
- Soil Cr was slightly above the normal level in the southwest region of China.

## 1. Introduction

Tea is among the three most popular non-alcoholic beverages worldwide [1]. It is mainly cultivated in Asia, Europe, East Africa, and South American countries, such as India, Kenya, China, Sri Lanka, and Turkey [1,2,3]. In 2019, world tea consumption was 5.859 million tons. Regular tea drinking is considered good for human health for beneficial tea components such as amino acids, tannins, polyphenols (catechins), and other antioxidants [4,5]. Furthermore, tea can supplement essential trace elements such as zinc, selenium, potassium, copper, boron, manganese, and strontium [6,7]. However, recently, there has been concern about heavy metals (HMs; arsenic (As), stibium (Sb), nickel (Ni), copper (Cu), thallium (Tl), mercury (Hg), cadmium (Cd), and chromium (Cr)) contaminating tea leaves from soils, especially Pb and Cd contamination [8]. The toxicities of HMs such as Cd, Cr, As, and Pb are recognized as major human risks worldwide [9]. Zhang et al. [10] reported Cd, Pb, and as pollution in tea plantation soil and Pb in tea leaves from Guizhou province, China; albeit, without serious risk to humans. Yaylalı-Abanuz et al. [11] reported Cd, Cu, and Hg contamination in the tea plantation soil of the mining area of eastern Black Sea in Turkey, with a relatively higher level of HMs in tea leaves compared to other countries.

Urbanization [12], mines [13], smelting [14], and agricultural activities [15] are the major anthropogenic pollution sources of HMs, whereas a high geochemical background can also naturally affect the soil HMs level [16]. Given the popularity and health benefits of tea, the risk assessment of HMs in tea plantation soil and leaves is crucial to ensure tea quality and safety [17]. However, the classic assessment methods, such as the single-factor index [18], potential ecological risk index [19], geo-accumulation [20], and Nemerow comprehensive index [21], have failed to integrate the HMs content in soil and agricultural products. Additionally, these ignore the problem of compound pollution, which often makes their assessment questionable. The comprehensive index method, which incorporates both soil and agricultural product contamination, can be a better approach for a close assessment of HMs pollution in farmland soil [22].

China has the largest tea plantation area and production in the world [10]. As the originator of tea trees, the southwest region of China had a tea planting area of 143.97 × 10^4^ ha^−1^ and a tea yield of 111.65 × 10^4^ t in 2020, accounting for 28.79% of the tea planting area and 18.15% of the tea yield worldwide, respectively. According to the atlas of soil environmental background value in the People’s Republic of China [23], the background values of the southwest region of China are 16.04, 0.240, 76.32, and 42.42 mg kg^−1^ for As, Cd, Cr and Pb, respectively, which are considered as a high HMs (As, Cd, Cr and Pb) geological background in China. Meanwhile, it is also affected by industrial activities, such as partial non-ferrous metal mining and selection, and agricultural activities involving pesticides and fertilizers for tea plantations [12,13,14,15,16]. In recent years, some tea plantation soils HMs (As, Cd, Cr and Pb) have exceeded the risk screening values for the soil contamination of agricultural land (GB15618-2018) in China, and therefore, excessive HMs in tea leaves may not be safe for human consumption [24]. Long-term consumption of HMs-enriched tea leaves may affect the nervous system, liver, and kidney [25]. Thus, the characteristics of HM pollution in the soil–tea system and potential health risks to humans are of increasing concern; accordingly, strict health assessments of the pollution risks are underway. For instance, the HMs assessment of tea leaves from a mining county of Fujian province, China, revealed that the personal total annual risk of oolong tea was slightly higher, while black and green tea were below the maximum acceptable levels of the US EPA (1.0 × 10^−4^) [26].Cao et al. [27] reported that the HMs (Pb, Al, Cd, Zn, As and Cu) HQ (hazard quotient) of raw and fermented Puerh tea from Puer in Yunnan, China, were <1, suggesting safe levels for tea consumption. Most of these studies mainly paid attention to clear sources of pollution, such as industrial, mining, smelting, and sewage irrigation, but only a few investigated HMs accumulation in the soil–tea systems of a superimposed high geochemical background area with human activities. The southwest region of China is a high HMs background area with relatively large tea plantation activity; however, the health risks related to the soil–tea system of this area are unknown.

Accordingly, this study aimed to evaluate the HMs (As, Cd, Cr and Pb) risk in the tea plantation soils and tea leaves from the southwest region of China using the Impact Index of Comprehensive Quality. The corresponding Cr, Cd, Pb, and As contents of soil and tea leaves were quantified and a potential relationship between the soil and tea leaves HMs content was investigated. Furthermore, the health risk assessment model (i.e., Target hazard quotient) was used to evaluate the health risk of long-term tea consumption in adults. These results may help to control HMs pollution in the test region.

## 2. Materials and Methods

### 2.1. Study Area

The study area (114,500 hm^2^), covering a total of 29 counties, is situated in the southwest region of China (97°31′~102°39′ E, 21°08′~25°52′ N). It mainly includes the three cities (prefectures) of Lincang City, Pu’er City, and Xishuangbanna Prefecture, along with some counties and districts of Baoshan City, Dali Prefecture, Honghe Prefecture, and Dehong Prefecture. The local climate is tropical and subtropical monsoon with an average annual temperature and precipitation of 19.5 °C and 1030.1 mm, respectively. The months from May to October are the peak rainy season. Lao’ai, Wuliang, and Gaoligong mountains are in the north of the study area with an elevation of <3000 m. The terrain gradually slows to the south and southwest with the broadening of the valley. In the south and southwest, the terrain gradually becomes gentle with the low mountains, wide valley basins, and elevation of about 800–1000 m; elevation in some areas drops to <500 m. Red soil is the main soil type. The southwest region is the major production area of tea in Yunnan Province, with a yield (dried tea) of 466,000 t in 2020, accounting for >90% of the whole province’s production.

### 2.2. Sample Collection

**Figure 1:**
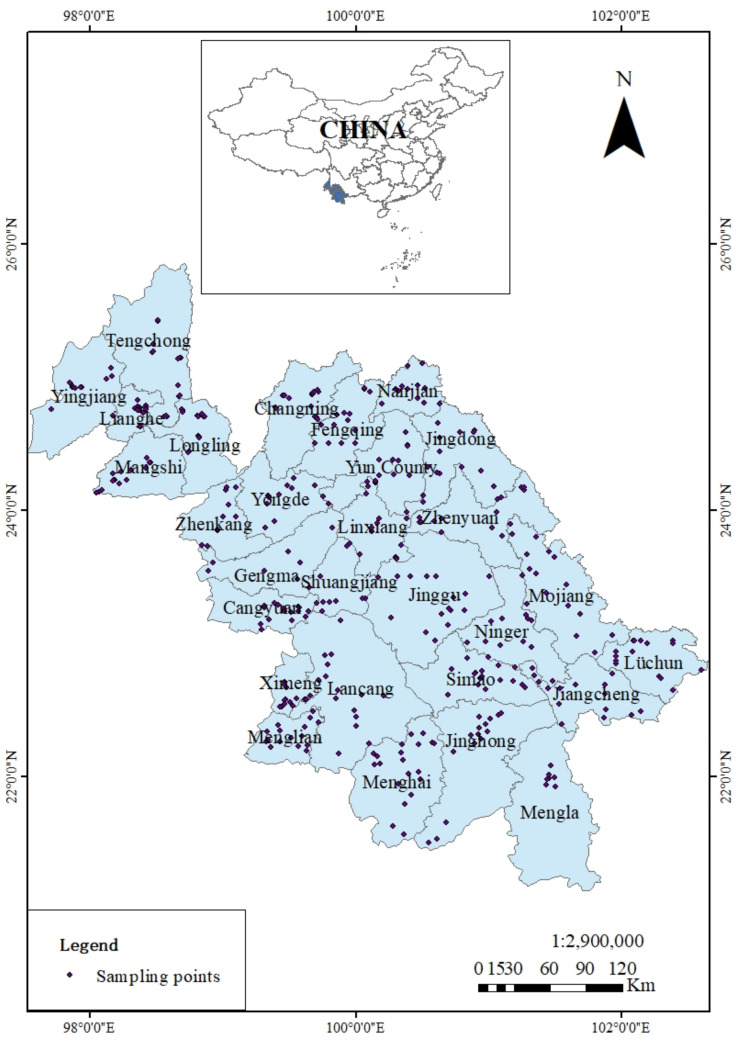
Map of the study area: the southwest region of Yunnan province, China.

Accounting for terrain and tea plantation distribution in southwest Yunnan, 421 pairs of soil and tea leaves samples were collected from 29 major tea-producing counties in the harvest season of September 2020. Each sample included five sub-samples within the 100 m^2^ of the main sample collection area. Soil samples, after being cleaned of plant roots, animal residues, and large rock fragments, were naturally air-dried, ground, and sieved at 2 mm. Composite samples were stored in a sample bottle until analysis. Meanwhile, the corresponding tea leaves samples (a bud and two leaves) were plucked randomly from each collection site. The tea leaves were rinsed with deionized water thrice, wiped dry, heated at 105 °C for 30 min, and then at 75 °C until they reached a constant weight. The dried tea leaves samples were ground and sieved through a 0.149-mm nylon sieve before storage at room temperature (RT) for later analysis. The specific sampling point information is presented in Figure 1.

### 2.3. Risk Assessment Method

Influence index of comprehensive quality (*IICQ*), combining the influence index of comprehensive quality for soil (*IICQ_s_*) and agricultural products (*IICQ_AP_*), takes into account the standard and valence effect of soil elements, the background value of soil elements, the limit standard of pollutants, and the content of target elements in agricultural products. It mainly comprises the following calculation process.

Determination of contaminating elements and quantities.

The measured values of elements in soil samples were compared with the evaluation standard and background values to find those that exceeded the limit, *X* and *Y*, respectively. Likewise, the estimated elemental values of agricultural products were compared with the pollutant limit standards for food to determine the Z-value of contaminants that exceeded the limit.

The *X* value for soil was calculated as follows:

(1) $$P_{ssi}=P_{i}/S_{i}$$

where *P_ssi_* is the index value of the sample element determination and evaluation criteria. For *P_ssi_* ≤ 1, take *x_i_* = 0; for *P_ssi_* > 1, take *x_i_* = 1; *X* is the sum of *x_i_*.

The *Y* value for soil was calculated as follows:

(2) $$P_{SBi}=C_{i}/C_{Bi}$$

where *P_SBi_* is the index value of the sample element determination value and the background value. For *P_SBi_* ≤ 1, take *y_i_* = 0; for *P_SBi_* > 1, take *y_i_* = 1; *Y* is the sum of *y_i_*.

The *Z* value for agricultural products was calculated as follows:

(3) $$P_{APi}=C_{APi}/C_{LSi}$$

where *P_APi_* is the index value of the determination value of elements in agricultural products and the limit value of pollutants in food. *C_APi_* is the concentration of element *i* in agricultural products, *C_LSi_* is the limit standard of element *i* in agricultural products. For *P_APi_* ≤ 1, take *z_i_* = 0; for *P_APi_* > 1, take *z_i_* = 1; *Z* value is the sum of *y_i_*.

Relative impact equivalent (*RIE*) was calculated as follows:

(4) $$RIE=\left[\overset{N}{\underset{i=1}{\sum }}{\left(P_{ssi}\right)}^{1/n}\right]/N=\left[\overset{N}{\underset{i=1}{\sum }}{\left(C_{i}/C_{si}\right)}^{1/n}\right]/N$$

where *N* is the number of elements, *C_i_* is the concentration of element *i*, *C_si_* is the standard value of soil environmental quality of element *i*; the Pb, Cr, As, and Cd standard values in China (NY/T853-2004) are 250, 150, 40, and 0.3 mg kg^−1^ for pH ≤ 6.5 soil, and 300, 200, 30, and 0.4 mg kg^−1^ for pH > 6.5 soil, respectively. *n* is the oxidation number of element *i* (Cr, 3; Pb, 2; Cd, 2; and As, 5). A larger *RIE* value suggests a bigger influence of exogenous substances.

The residue limits of tea leaves for Pb, Cr, As, and Cd (NY695-2003 and GB2762-2017) were 5, 5, 2, and 1 mg kg^−1^, respectively.

The deviation degree of determination concentration from the background value (*DDDB*) was calculated as follows:

(5) $$DDDB=\left[\overset{N}{\underset{i=1}{\sum }}{\left(P_{SBi}\right)}^{1/n}\right]/N=\left[\overset{N}{\underset{i=1}{\sum }}{\left(C_{i}/C_{Bi}\right)}^{1/n}\right]/N$$

where *C_Bi_* is the background value of element *i* (shown in Table 1), and the other symbols remain the same as above. A larger *DDDB* value suggests a greater influence of exogenous substances.

The deviation degree of standard from the background value (*DDSB*) was calculated as follows:

(6) $$DDSB=\left[\overset{N}{\underset{i=1}{\sum }}{\left(C_{Si}/C_{Bi}\right)}^{1/n}\right]$$

Symbols stay the same as above. A larger *DDDB* value suggests a greater deviation of soil standard from the background value with the greater load capacity of the soil and stronger buffer to the exogenous substances.

The quality index of agricultural products (*QIAP*) was calculated as follows:

(7) $$QIAP=\left[\overset{N}{\underset{i=1}{\sum }}{\left(P_{APi}\right)}^{1/n}\right]/N=\left[\overset{N}{\underset{i=1}{\sum }}{\left(C_{APi}/C_{LSi}\right)}^{1/n}\right]/N$$

where *C_APi_* is the concentration of element *i* in agricultural products, *C_LSi_* is the residue limit for element *i* in agricultural products. The residue limits of tea leaves for Cd, Cr, Pb, As (NY695-2003 and GB2762-2017) are 1, 5, 5, and 2 mg kg^−1^, respectively. *QIAP* indicates the influence of HMs on the quality of agricultural products. A higher residue limit value of HMs in agricultural products suggests the worsening of quality.

The influence index of comprehensive quality (*IICQ*) was calculated as follows.

(8) $$IICQ_{S}=X\times \left(1+RIE\right)+Y\times \left(DDDB\right)/DDSB$$

(9) $$IICQ_{AP}=Z\times \left(1+QIAP/k\right)+QIAP/\left(k\times DDSB\right)$$

Namely,

(10) $$IICQ=IICQ_{S}+IICQ_{AP}$$

(11) $$\begin{array}{c} =\left[X\times (1+RIE)+Y\times (DDDB)/DDSB\right]+[Z\times \left(1+QIAP/k\right)\\ +QIAP/\left(k\times DDSB\right) \end{array}]$$

where *IICQ_s_* and *IICQ_AP_* are the influence index of comprehensive quality for soil and agricultural products, respectively. *X* and *Y* are the numbers of measured values that exceeded soil standard and background values, respectively. *Z* is the quantities of measured values that exceeded the threshold limit of agricultural products. *k* is the correction parameter of background, which was set to 5 for food safety.

Based on the sum of *IICQ_s_* and *IICQ_AP_* in a region, the soil–tea system HMs pollution risk was categorized into the following 5 grades: <1, uncontaminated; 1–2, slightly contaminated; 2–3, moderately contaminated; 3–5, heavily contaminated, and above 5, extremely contaminated.

### 2.4. Health Risk Assessment Method

Target hazard quotient (*THQ*) was established by the United States Environmental Protection Agency (EPA) in 2000 to assess the non-carcinogenic risk of human exposure to pollutants such as HMs. It assumes the absorbed dose of pollutants equal to the intake dose and uses the ratio of human absorbed dose of pollutants to the reference dose for evaluation. Here, we only calculated the exposure risk of tea intake and did not consider any other routes of exposure. *THQ* was calculated using the following formula:

(12) $$THQ=\frac{E_{F}\times E_{D}\times F_{IR}\times c}{Rfd\times W_{AB}\times T_{A}}\times 10^{-3}$$

where *E_F_* is the exposure frequency in adults, 350 d a^−1^; *E_D_* is the exposure duration, 24 a; *F_IR_* is the daily tea intake rate, 0.0094 kg d^−1^; c is the average content of HMs in tea, mg kg^−1^; *Rfd* is the daily allowance for HMs such as 0.0035, 0.001, 0.003 and 0.0003 for lead, cadmium, chromium, and arsenic, respectively. *W_AB_* is the average weight of an adult, 56.8 kg. *T_A_* is the average exposure time (set to 350 days × 24 years) for non-carcinogens. A *THQ* value less than 1 indicates an acceptable risk of HMs exposure from tea; on the contrary, a higher *THQ* value suggests increased risk to human health.

The impact of HMs on human health is a combined action of multiple elements; the composite risk of multiple heavy metals was calculated using the following formula:

(13) $$HI=\sum THQ\left(\mathrm{sing}\;\mathrm{pollutant}\right)$$

where *HI* is the hazard index. *HI* < 1, *HI* > 1, and *HI* > 10 indicate no obvious negative impact, a high possibility of negative effects, and chronic toxicity, respectively.

### 2.5. Sample Analysis

Soil pH was determined as per the glass electrode method (liquid comprised of 1:2.5 *w*/*v*) [28], and the soil organic matter was determined as per the external heating method using potassium dichromate [28]. Soil HMs (Pb, Cr, As, and Cd) were digested with concentrated nitric, hydrochloric, and hydrofluoric acids using a microwave digestion apparatus (Multiwave 5000, Anton Paar, Austria) following the HJ832-2017 method in China. Pb and Cd in digested soil solution were determined using Graphite furnace atomic absorption spectrophotometry (GFAAS, PerkinElmer 900H, PerkinElmer, Akron, OH, USA). As and Cr were determined using atomic fluorescence spectrometry (AFS-933, Titan Instrument, Beijing, China) and flame atomic absorption spectrophotometry (FAAS, PerkinElmer 900H, PerkinElmer, Akron, OH, USA), respectively.

HMs Pb, As, and Cr in tea leaves samples were digested with concentrated nitric acid using a microwave digestion apparatus (Multiwave 5000, Anton Paar, Austria) following the respective GB5009.12, GB 5009.11, and GB 5009.123 methods in China. Cd in tea leaves was digested with concentrated nitric acid and 30% H_2_O_2_ according to the GB5009.15 method in China. The Cr, Cd, and Pb in the digested liquids were measured utilizing GFAAS, while As was measured using inductively coupled plasma mass spectrometry (ICP-MS, Qc, Thermo Fisher, Waltham, MA, USA).

For the soil sample analytical method, the limits of detection values (LODs) for Pb, Cr, As, and Cd were 0.1, 4.0, 0.01, 0.01 mg kg^−1^, respectively. Likewise, for the tea leaves sample determination method, LODs for Pb, Cr, As, and Cd were 0.02, 0.01, 0.002, and 0.001 mg kg^−1^, respectively. Furthermore, a certified reference soil (GBW07451) and tea leaves material (GBW08513) from the National Research Center for standard materials in China were used for quality control of the sample batch. The concentrations of measured reference materials were within the certified ranges; the average recoveries for Pb, Cr, As, and Cd were 98.2, 103.0, 92.5, and 93.5% for soils, and 94.7, 104.0, 101.0, and 98.2% for tea leaves, respectively. All determinations were conducted thrice to ensure the data quality.

### 2.6. Data Analysis

All statistical procedures were performed using the Origin 9.0 (Origin Lab Corporation, Northampton, MA, USA) and Excel 2007 (Microsoft, Redmond, WA, USA) programs. The spatial distribution map and Kriging interpolation map were prepared using ArcGIS10.2 (Esri, Redlands, CA, USA). Before the data analysis, survey data were checked for normal distribution.

## 3. Results

### 3.1. Heavy Metals in Soils and Tea Leaves

**Figure 2:**
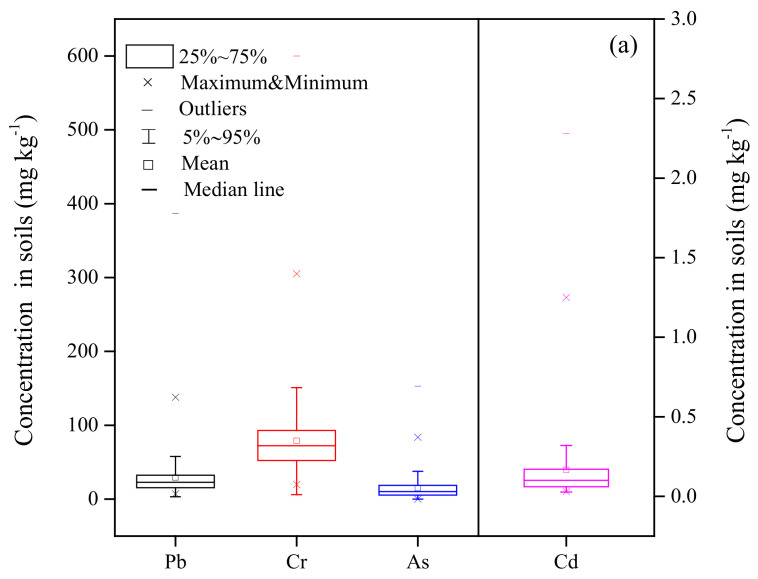

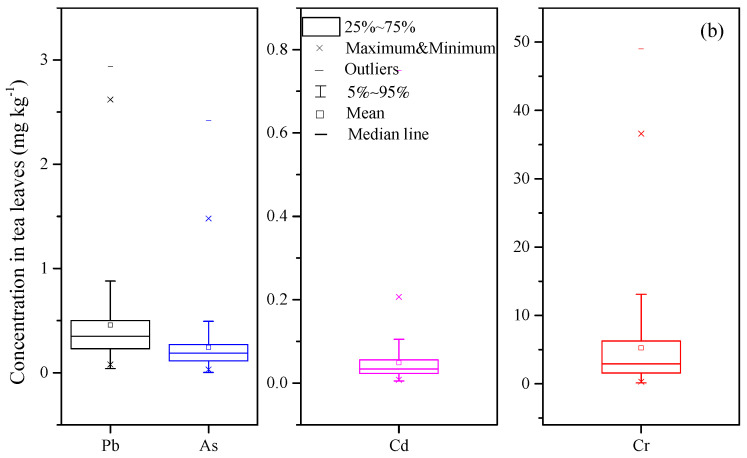
Contents of Pb, Cr, As, and Cd in soil (**a**) and tea leaves (**b**) samples. The standard values in China (NY/T853-2004) for Pb, Cr, As, and Cd are 250, 150, 40, and 0.3 mg kg^−1^ for pH ≤ 6.5 soil, and 300, 200, 30, and 0.4 mg kg^−1^ for pH > 6.5 soil. The residue limits values of tea leaves (NY695-2003 and GB2762-2017) for Pb, Cr, As, and Cd are 5, 5, 2, and 1 mg kg^−1^, respectively.

Descriptive statistics for soil HMs contents from the southwest region of Yunnan province, China, are shown in Figure 2a. The average amounts of Pb (3.2 to 387 mg kg^−1^), Cd (0.03 to 7.94 mg kg^−1^), Cr (6 to 600 mg kg^−1^), and As (0.01 to 153 mg kg^−1^) were 29.27, 0.18, 79.06, 14.87 mg kg^−1^ (*n* = 421), respectively; the numbers in parentheses show the range. Additionally, 0.71, 10.21, 4.99, and 7.36% of soil samples exceeded the threshold values (NY/T 853-2004) of Pb, Cd, Cr, and As, respectively.

Likewise, the HMs contents in the tea leaves from the southwest region of Yunnan province, China, are presented in Figure 2b. The average amounts of Pb (0.04 to 2.90 mg kg^−1^), Cd (0.005 to 0.620 mg kg^−1^), Cr (0.13 to 49.0 mg kg^−1^), and as (0.001 to 2.42 mg kg^−1^) were 0.46, 0.05, 5.28, 0.25 mg kg^−1^ (*n* = 421), respectively; the numbers in parentheses show the range. Pb, As and Cd were below the set limits, while Cr exceeded the amounts set by the Ministry of Agriculture of China (NY659-2003 and GB2762-2017).

### 3.2. Correlation Analysis

**Figure 3:**
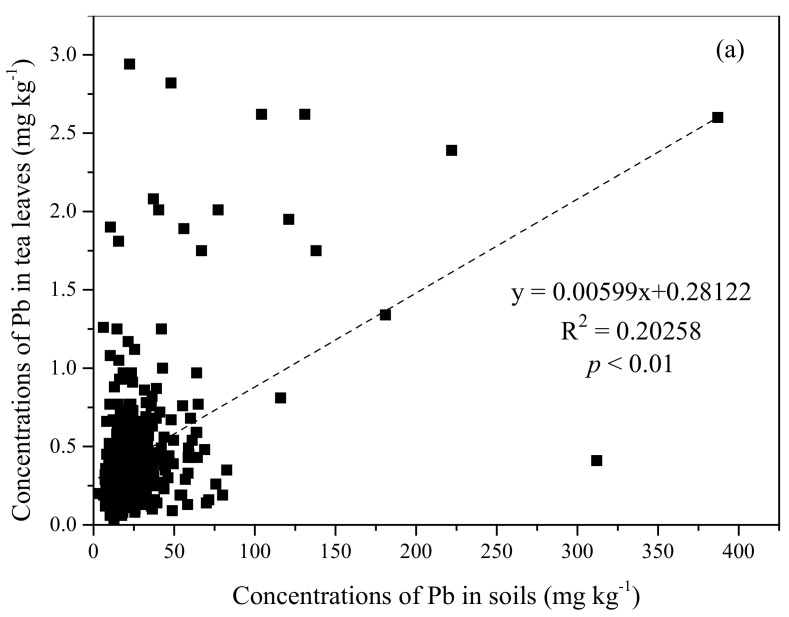

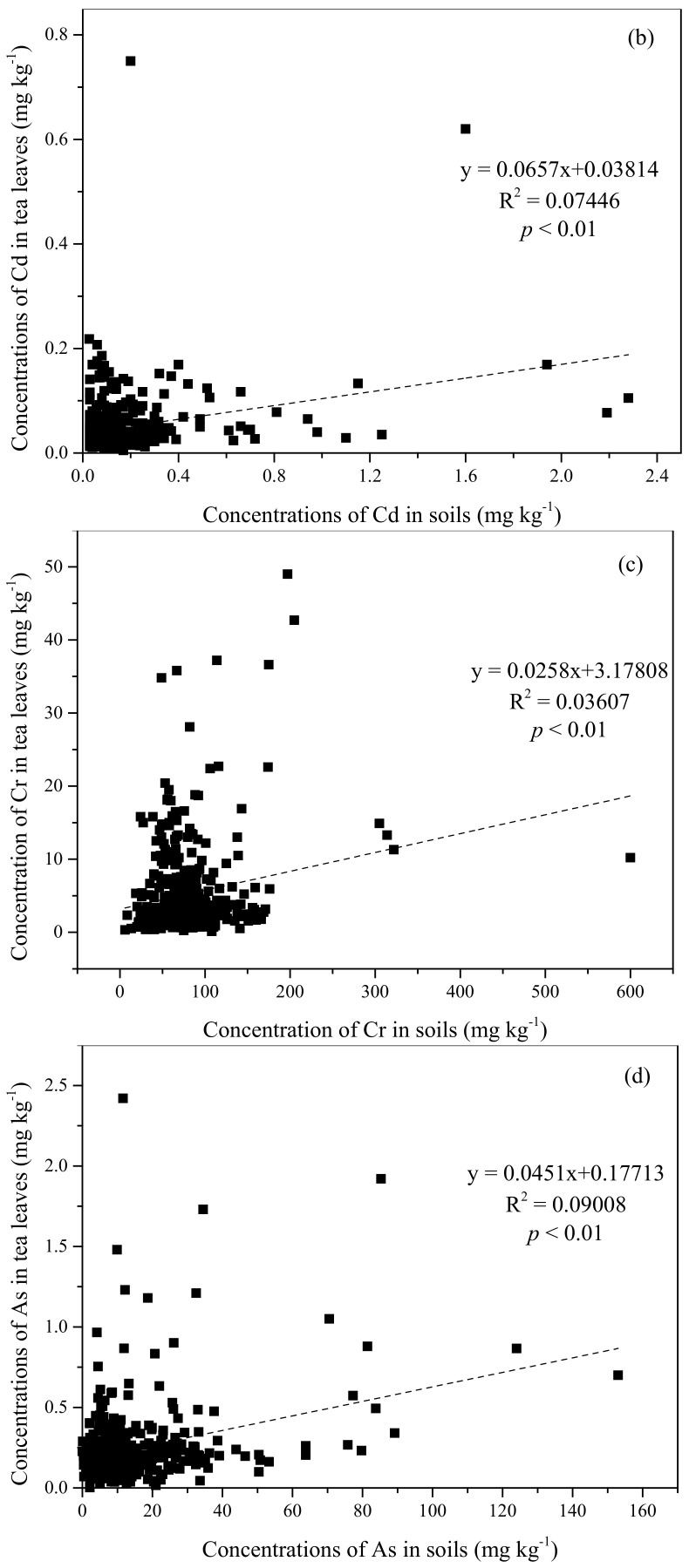
The relationship between the concentrations of heavy metals (i.e., (**a**) Pb, (**b**) Cd, (**c**) Cr, and (**d**) As) in soil and corresponding tea leaves. Sample size = 421.

The relationship between the total concentrations of HMs in soil and tea leaves is shown in Figure 3. The amounts of Pb, Cr, As, and Cd in the tea leaves positively correlated with their amounts in the soil (*p* < 0.01) with an R^2^ of 0.203 **, 0.074 **, 0.036 **, 0.090 **, respectively. Further statistical analysis revealed that the correlations between the soil and tea leaves HMs amounts were as follows: Pb > As > Cd > Cr. This suggests that the increase in the total HMs concentration in the soil has a certain effect on the absorption and accumulation of HMs in tea leaves, but it is not the main factor.

### 3.3. Collaborative Assessment

A collaborative assessment of the HMs in soils and tea leaves from the southwest region of Yunnan province is shown in Table S1. Among the 421 collected samples, 80.05, 6.18, 10.69, 1.19, and 1.90% of the soil samples were found as uncontaminated, slightly contaminated, moderately contaminated, heavily contaminated, and extremely contaminated, respectively. Correspondingly, 71.50% of the tea leaves samples were contaminated; only 28.03 and 0.48% of the tea leaves samples were slightly or moderately contaminated.

**Figure 4:**
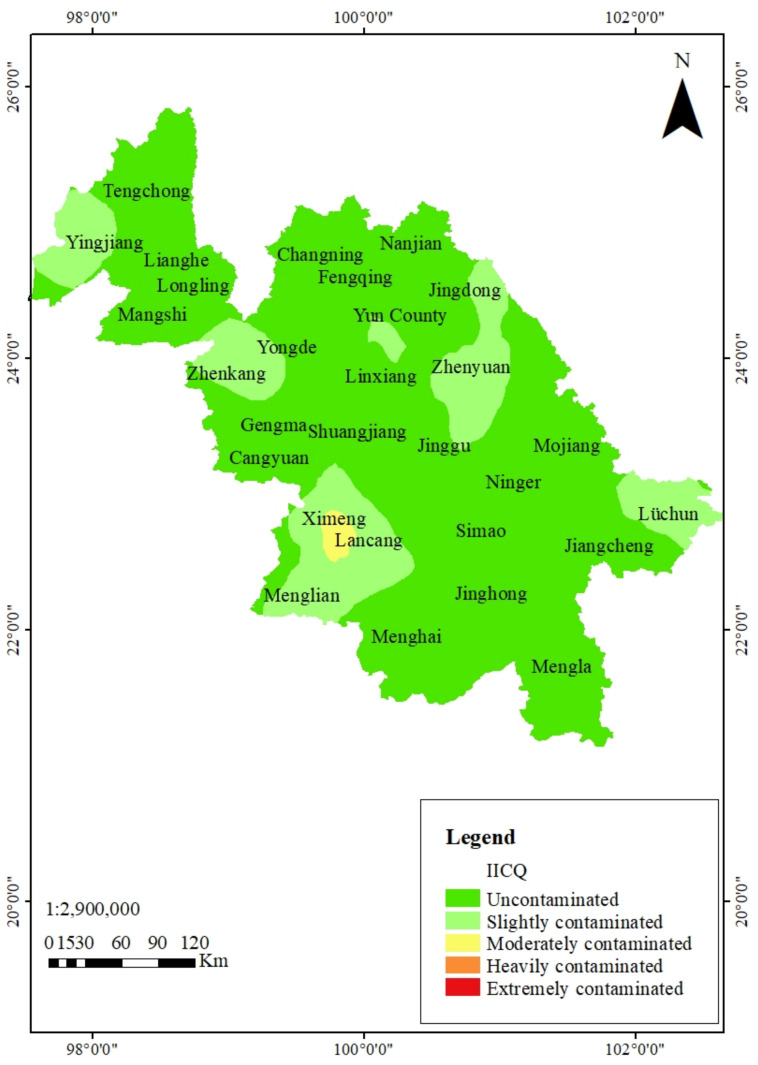
Prediction map of the potential risk of heavy metal contamination in tea plantation areas based on the collaborative assessment. *IICQ* denotes the overall quality of soil and tea leaves divided into 5 grades: below 1, uncontaminated; 1–2, slightly contaminated; 2–3, moderately contaminated; 3–5, heavily contaminated; and >5, extremely contaminated.

The Influence Index of Comprehensive Quality (*IICQ*), the collaborative assessment of HMs in soils and tea leaves, broadly considers soil quality and food safety (Table S1). We found that 59.62, 24.47, 7.60, 5.70, and 2.61% of the samples were uncontaminated, slightly contaminated, moderately contaminated, heavily contaminated, and extremely contaminated, respectively. A spatial distribution map of the collaborative assessment of HMs was created using geostatistical methods, ordinary Kriging in GIS (Figure 4). We found that the moderately contaminated (high *IICQ* blocks; yellow color) samples were from Lancang County, the southwest of the study region, while the slightly contaminated (light green color blocks) were scattered to Yingjiang, Zhenkang, Yongde, Zhenyuan, Lüchun, Jingdong, Ximeng, and Menglian.

### 3.4. Health Risk Assessment

The target hazard quotient method was used to assess the health risk associated with the consumption of HMs-contaminated tea leaves in adults (Table 2). The corresponding *THQ* values for Pb, Cr, As, and Cd were 0.021, 0.288, 0.136, and 0.007, respectively. Based on the mean THQ, the order of HMs contamination was Cr > As > Pb > Cd; the risk index was less than one. This suggests that exposure to a single HM via tea consumption is not a potential health risk to adults.

The *HI* is a useful parameter to evaluate overall potential health risk from multiple HMs consumption. The *HI* values, with an average value of 0.452 (range: 0.159 to 0.980), from all the southwest region counties of Yunnan province, China, were less than unity. This suggests that the HMs from tea consumption were not a non-carcinogenic risk.

## 4. Discussion

### 4.1. HMs Contents in the Soil–Tea System

The HMs enrichment of tea plantation soils can be from both natural (e.g., high soil HM geochemical background) and human sources (e.g., mining activities, application of chemical fertilizers, and pesticides containing HMs) [12,13,14,15,16]. In this study, we found that the environmental quality of tea plantation soils from the southwest region of Yunnan province, China, was largely good; however, 0.71, 4.99, 7.36, and 10.21% of the soil samples exceeded the threshold values (NY/T 853-2004) for Pb, Cr, As, and Cd, respectively. This indicated a soil HMs accumulation in some regions. The average HMs content in tea plantation soil was compared to different previously reported locations worldwide (Table S1). The mean concentrations of Cr, As, Cd were slightly higher, while the Pb amounts were in the moderate range. The high soil geochemical background of Cr, As and Cd in the southwest region of Yunnan province is the most significant factor to soil HMs contamination.

The HMs enrichment of tea leaves is a complicated process affected by the soil HMs content, the bioconcentration coefficient of tea leaves, and the soil physic-chemical properties [17]. In our case, the soil HMs content appears to be an important factor; notably, it showed a positive correlation with the corresponding HMs in tea leaves at *p* < 0.01 levels. This is consistent with Zhang et al. [10] suggesting Cd amounts in tea leaves were linked to Cd content in tea plantation soils. Other studies reported similar findings for other metals, such as Mn, Cr, Se, and Tl [10,12]. For example, Li et al. [29] reported that Se content in rice seeds was weakly positively correlated with the total Se content in the corresponding root-soil (R^2^ 0.0435; *p* < 0.01). In our study, the average values (mg kg^−1^) of Pb (0.46), Cd (0.05), and As (0.25) were below the corresponding residue limits, while Cr (5.28) was above the prescribed limits. This indicated that the HMs in the tea leaves were overall in a safe range. A higher Cr content in the tea leaves may be linked to its relatively high abundance in the soils (6 to 600 mg kg^−1^) compared to other reported locations worldwide. In addition, the dustfall, with HMs increasing tea leaves absorption through wet–dry atmospheric deposition, can also be a potential pollution source [30]. Thus, it is necessary to assess in detail the severity of soil contamination and tea leaves safety in the southwest region of China.

### 4.2. Comprehensive Evaluation of the Overall Quality of Soils and Tea Leaves

In this study, we used *IICQ* to assess the impacts of HMs contamination in the soil–tea system [22]. It reflects the interaction between soil and agricultural product heavy metal for farmland soil environmental quality, while considering the ion impulse and HM loading capacity of the soil. Compared with classic evaluation methods, including the single factor index [18], the potential ecological risk index [19], geo-accumulation [20], and the Nemerow comprehensive index [21], it is more objective and reliable. Based on *IICQ* analysis, we found that 59.62, 24.47, 7.60, 5.70, and 2.61% of the samples were uncontaminated, slightly contaminated, moderately contaminated, heavily contaminated, and extremely contaminated, respectively. This indicated that the environmental quality of soils and tea leaves from the southwest region of Yunnan province, China, was generally well, while ~40.38% of the plantation area was contaminated by one or more HM(s). The contaminated areas need urgent remediation due to the severe safety risk of tea leaves, and human health.

Moreover, the spatial distribution map of the collaborative assessment of HMs revealed moderate contamination in Lancang County in the southwest of the study region, while Yingjiang, Zhenkang, Yongde, Zhenyuan, Lüchun, Jingdong, Ximeng, and Menglian were slightly contaminated. There could be several possible explanations for this spatial distribution of HMs. Firstly, Lancang County, which is rich in >30 kinds of metal and non-metallic minerals such as iron, Pb-Zn, lignite, etc., shows high tea leaves enrichment of Pb, Cd, and As, possibly from their high soil amounts due to the mining, smelting, and rolling of Pb–Zn mining. Second, the slight and moderate levels of pollution could be from the application of chemical fertilizers and pesticides, which leads to HMs enrichment in tea leaves. The third possible reason could be high mean geochemical background values (mg kg^−^^1^) for Pb (36.12), Cr (63.04), As (9.69), and Cd (0.09), respectively [23]. Therefore, both high geochemical background and human activities can be contributors to the HMs contamination of tea plantation soils.

### 4.3. Risk of Adult Exposure to Drinking Tea

Soil HMs can reach into tea trees through the root system and accumulate in different organs and tissues [5]. A large accumulation of HMs in tea leaves [31,32] can endanger human health through tea drinking [33]. The toxicities of Pb, Cd, Cr, and As are recognized as major human health risks worldwide [34,35]. With increasing health awareness, the HM contamination in tea leaves has emerged as a great concern. It is unknown whether tea planting areas with a high heavy metal background will pose a threat to human health. In this study, we found that the health risk index for a single heavy metal (Pb, Cd, Cr, and As) was less than unity in adults, suggesting safe levels. The mean *THQ* of different metals were Cr > As > Pb > Cd, which indicated that Cr contamination, with 6.24 to 96.17% of TTHQ, was the major health risk. The is in line with Wang et al. [26] showing Cr in tea leaves was the major health risk in a mining county of central Fujian province. Other studies reported similar findings for Pu’er and black tea [27,36]. Therefore, Cr contamination in tea leaves needs special attention and prevention measures, such as spraying leaf tissue control agents to reduce Cr absorption in tea leaves [37] and finding tea varieties with a reduced risk of Cr accumulation [26].

In this study, for the southwest region of Yunnan province, China, the hazard index (*HI*) for HMs ranged from 0.159 to 0.980, with an average value of 0.452; all the counties had *HI* values less than unity. This suggested no non-carcinogenic risk for adults from tea leaves consumption. A study conducted in the high geological background (for Tl, Cd, As, Sb, Pb, Cr, Hg, and Ni) of Guizhou province also found a *HI* below unity [10]. Additionally, Shen et al. [4] reported that the *HI* of the daily consumption of black tea, oolong tea, and green tea was low without any health risks. Although the health risk of HMs ingestion via tea leaves is relatively low, there could be other pathways that may expose HMs to humans. Therefore, more comprehensive investigations are needed for a health risk assessment.

## 5. Conclusions

We found that 0.71, 4.99, 7.36, and 10.21% of the soil samples from the southwest region of Yunnan province, China, exceeded the threshold values (NY/T 853-2004) for Pb, Cr, As, and Cd, respectively. The average concentrations of Pb, As, and Cd in tea leaves were less than the corresponding residue limits (NY659-2003 and GB2762-2017), but the mean concentration of Cr was above the corresponding threshold. Correlation analysis revealed that the amounts of Pb, Cr, As, and Cd in the tea leaves positively correlated with the corresponding soil amounts. The collaborative assessment indicated that the overall environmental quality of soils and tea leaves from the southwest area was good, while ~40.38% of the area was contaminated by one more HM. Furthermore, spatial distribution analysis revealed that Lancang was moderately contaminated, while Yingjiang, Zhenkang, Yongde, Zhenyuan, Lüchun, Jingdong, Ximeng, and Menglian were slightly contaminated regions. The *THQ* of Pb, Cr, As, and Cd, and the *HI* indicated no non-carcinogenic risk to adults from tea consumption. Our data may help to control the HMs pollution in tea plantation soil to ensure the quality and safety of tea leaves in the test region.

## Figures and Tables

**Table 1:** Background values of lead (Pb), chromium (Cr), arsenic (As), and cadmium (Cd) in soils from selected regions.

Region	Pb	Cd	Cr	As
	mg kg^−1^			
Tengchong	39.16	0.139	58.8	7.42
Longling	39.16	0.139	58.8	7.42
Changning	39.16	0.139	58.8	7.42
Lianghe	43.82	0.071	55.2	4.25
Yingjiang	43.82	0.071	55.2	4.25
Mangshi	43.82	0.071	55.2	4.25
Fengqing	39.67	0.115	75.7	13.47
Yongde	39.67	0.115	75.7	13.47
Zhenkang	39.67	0.115	75.7	13.47
Linxiang	39.67	0.115	75.7	13.47
Yun County	39.67	0.115	75.7	13.47
Gengma	39.67	0.115	75.7	13.47
Cangyuan	39.67	0.115	75.7	13.47
Shuangjiang	39.67	0.115	75.7	13.47
Nanjian	34.16	0.128	68.7	8.39
Jingdong	32.83	0.060	59.0	8.77
Zhenyuan	32.83	0.060	59.0	8.77
Jinggu	32.83	0.060	59.0	8.77
Lancang	32.83	0.060	59.0	8.77
Ximeng	32.83	0.060	59.0	8.77
Menglian	32.83	0.060	59.0	8.77
Mojiang	32.83	0.060	59.0	8.77
Jiangcheng	32.83	0.060	59.0	8.77
Ninger	32.83	0.060	59.0	8.77
Simao	32.83	0.060	59.0	8.77
Menghai	25.80	0.061	49.8	9.15
Jinghong	25.80	0.061	49.8	9.15
Mengla	25.80	0.061	49.8	9.15
Lüchun	41.24	0.099	72.4	14.65

**Table 2:** The calculated target hazard quotients (*THQ*) of Pb, Cd, Cr, and As, and hazard index (*HI*) for adults associated with the tea leaves consumption in the southwest region of Yunnan province, China.

Region	Risk Assessment Index				
	*THQ*_Pb_	*THQ*_Cd_	*THQ*_Cr_	*THQ*_As_	*HI*
Tengchong	0.020	0.002	0.044	0.093	0.159
Longling	0.020	0.005	0.175	0.098	0.298
Changning	0.018	0.004	0.200	0.134	0.356
Lianghe	0.029	0.003	0.090	0.145	0.268
Yingjiang	0.014	0.002	0.135	0.168	0.319
Mangshi	0.017	0.006	0.242	0.152	0.416
Fengqing	0.020	0.007	0.141	0.083	0.251
Yongde	0.016	0.010	0.298	0.083	0.407
Zhenkang	0.013	0.022	0.794	0.152	0.980
Linxiang	0.024	0.009	0.196	0.118	0.347
Yun County	0.039	0.009	0.108	0.153	0.308
Gengma	0.011	0.013	0.065	0.054	0.143
Cangyuan	0.019	0.008	0.251	0.072	0.350
Shuangjiang	0.020	0.008	0.128	0.104	0.260
Nanjian	0.019	0.004	0.435	0.140	0.599
Jingdong	0.012	0.011	0.209	0.109	0.341
Zhenyuan	0.033	0.010	0.548	0.195	0.786
Jinggu	0.011	0.005	0.215	0.138	0.369
Lancang	0.040	0.010	0.538	0.225	0.814
Ximeng	0.019	0.004	0.538	0.170	0.730
Menglian	0.012	0.009	0.523	0.165	0.710
Mojiang	0.034	0.009	0.259	0.222	0.523
Jiangcheng	0.015	0.005	0.287	0.083	0.390
Ninger	0.012	0.013	0.165	0.079	0.269
Simao	0.016	0.006	0.274	0.133	0.429
Menghai	0.027	0.005	0.583	0.255	0.870
Jinghong	0.015	0.005	0.350	0.084	0.453
Mengla	0.024	0.006	0.191	0.214	0.435
Lüchun	0.029	0.007	0.217	0.086	0.338
Study area	0.021	0.007	0.288	0.136	0.452

Note: *THQ*_Pb_, *THQ*_Cd_, *THQ*_Cr_, *THQ*_As_ are the target hazard quotients values of Pb, Cd, Cr, and As, respectively; and *HI* is the hazard index values.

## Data Availability

Not applicable.

## Supplementary Materials

The following are available online at https://www.mdpi.com/article/10.3390/ijerph181910151/s1, Table S1: The concentration of heavy metals in tea plantation soil from different areas. Table S2: The calculation of parameter for collaborative assessment. Table showing the concentration of heavy metals in tea plantation soil from the different areas; table showing the parameters of collaborative assessment.
