# Supplementary material for: Collaborative Assessment and Health Risk of Heavy Metals in Soils and Tea Leaves in the Southwest Region of China
Source: Int J Environ Res Public Health. 2021 Sep 27;18(19):10151. doi: 10.3390/ijerph181910151 (PMC8508298; doi:10.3390/ijerph181910151)
Supplement: Supplementary file 1 [file ijerph-18-10151-s001.zip › ijerph-1356444-supplementary.pdf]

SUPPLEMENTAL MATERIAL

Table showing the concentration of heavy metals in tea plantation soil from the different areas; table showing the parameters of collaborative assessment.

Table S1 The concentration of heavy metals in tea plantation soil from different areas

Region	Pb	As	Cd	Cr	Reference
	mg kg^{-1}				
Guizhou, China	67.1	22.8	0.47	77.9	Zhang et al.,2013
Guizhou, China	40.9	26.3	0.33	66	Zhang et al.,2020
Jiangsu, China	34.65	-	-	84.74	Wen et al.,2018
Guangdong, China	14.4	-	0.063	29.3	Li et al.,2010
Shandong, China	34.1	5.79	0.16	47.6	Hu et al.,2009
Sichuan, China	12.0	6.52	0.072	25.6	Tan et al.,2005
Hunan, China	42.7	12.4	0.074	92.2	Guo et al.,2008
Anhui, China	8.5	68.0	-	51.8	Fang et al.,2015
Anhui, China	45.77	-	0.76	-	Peng et al.,2018
Jiangxi, China	24.6	21.8	0.133	64.7	Xiong et al.,2011
Fujian(a mine county), China	78.0	50.7	0.23	110	Wang et al.,2018
Fujian(Wuyi moutian) , China	76.5	6.13	0.19	67.5	Ye et al.,2016
Fujian(Anxi) , China	48.3	7.37	0.036	53.0	Guo et al.,2011
Gilan and Mazandaran provinces , Iran	11.43	-	0.36	-	Mohammad et al.,2014
Hubei, China	23.1	8.91	0.11	50.6	Wang et al.,2010
Shanxi, China	-	-	-	47.0	Zhao et al.,2016
Study area	29.27	14.87	0.18	79.06	This study

Table S2. The calculation of parameter for collaborative assessment.

NO.	Soil					Agricultural product		Hyper-standard,X	Hyper-standard,Y	Hyper-standard,Z	IICQ
	pH	RIE	DDDB	DDSB	$IICQ_S$	QIAP	$IICQ_{AP}$				
1	6.60	0.794	1.331	7.264	2.344	0.609	0.017	1	3	0	2.361
2	7.18	0.815	1.392	7.264	2.390	0.451	0.012	1	3	0	2.402
3	4.17	0.647	0.979	6.700	0.438	0.441	0.013	0	3	0	0.452
4	4.03	0.643	0.966	6.700	0.433	0.408	0.012	0	3	0	0.445

5	4.03	0.713	1.112	6.700	2.045	0.409	0.012	1	2	0	2.057
6	4.17	0.712	1.113	6.700	2.044	0.427	0.013	1	2	0	2.057
7	5.05	0.755	1.138	6.700	2.434	0.509	0.015	1	4	0	2.450
8	5.13	0.740	1.114	6.700	2.239	0.528	0.016	1	3	0	2.255
9	4.56	0.701	1.071	6.700	0.320	0.417	0.012	0	2	0	0.332
10	4.76	0.748	1.155	6.700	2.092	0.402	0.012	1	2	0	2.104
11	4.72	0.847	1.387	6.700	0.828	0.605	1.139	0	4	1	1.967
12	6.53	0.912	1.591	7.264	4.700	0.623	1.142	2	4	1	5.842
13	5.21	0.670	1.066	6.700	0.318	0.469	0.014	0	2	0	0.332
14	4.84	0.541	0.831	6.700	0.000	0.583	1.134	0	0	1	1.134
15	4.72	0.548	0.842	6.700	0.000	0.561	1.129	0	0	1	1.129
16	4.75	0.559	0.865	6.700	0.000	0.508	1.117	0	0	1	1.117
17	5.55	0.401	0.593	6.899	0.086	0.428	0.012	0	1	0	0.098
18	5.39	0.566	0.880	6.899	0.000	0.620	1.142	0	0	1	1.142
19	5.65	0.679	1.048	6.899	0.456	0.691	1.158	0	3	1	1.614
20	7.47	0.632	1.039	7.448	0.419	0.403	0.011	0	3	0	0.429
21	6.65	0.562	0.915	7.448	0.123	0.367	0.010	0	1	0	0.133
22	7.78	0.679	1.134	7.448	0.305	0.464	0.012	0	2	0	0.317
23	7.18	0.801	1.365	7.448	1.984	0.678	1.154	1	1	1	3.138
24	6.84	0.574	0.942	7.448	0.126	0.660	1.150	0	1	1	1.276
25	7.03	0.593	0.970	7.448	0.130	0.678	1.154	0	1	1	1.284
26	7.64	0.778	1.315	7.448	2.131	0.671	1.152	1	2	1	3.284
27	5.53	0.631	0.981	6.899	0.285	0.496	0.014	0	2	0	0.299
28	7.80	0.787	1.335	7.448	2.146	0.651	1.148	1	2	1	3.293
29	6.37	0.660	1.076	7.136	0.603	0.421	0.012	0	4	0	0.615
30	6.51	0.645	1.070	7.448	0.431	0.463	0.012	0	3	0	0.444
31	5.63	0.602	0.935	6.899	0.271	0.424	0.012	0	2	0	0.283
32	5.61	0.718	1.173	6.899	0.680	0.373	0.011	0	4	0	0.691
33	4.91	0.566	0.916	6.763	0.271	0.364	0.011	0	2	0	0.282
34	5.08	0.560	0.878	6.763	0.130	0.398	0.012	0	1	0	0.142
35	5.14	0.587	0.952	6.763	0.282	0.300	0.009	0	2	0	0.290

36	4.83	0.573	0.923	6.763	0.273	0.361	0.011	0	2	0	0.284
37	4.57	0.562	0.899	6.763	0.000	0.252	0.007	0	0	0	0.007
38	4.42	0.554	0.979	7.371	0.266	0.279	0.008	0	2	0	0.273
39	4.95	0.612	0.980	6.763	0.290	0.229	0.007	0	2	0	0.297
40	4.85	0.565	0.903	6.763	0.000	0.525	0.016	0	0	0	0.016
41	5.33	0.465	0.752	6.763	0.000	0.265	0.008	0	0	0	0.008
42	5.24	0.568	0.989	7.371	0.268	0.285	0.008	0	2	0	0.276
43	4.89	0.525	0.846	6.763	0.000	0.302	0.009	0	0	0	0.009
44	4.80	0.538	0.943	7.371	0.256	0.327	0.009	0	2	0	0.265
45	5.20	0.488	0.787	6.763	0.000	0.349	0.010	0	0	0	0.010
46	4.85	0.736	1.174	6.763	0.695	0.336	0.010	0	4	0	0.705
47	5.15	0.608	0.989	6.763	0.292	0.498	0.015	0	2	0	0.307
48	4.54	0.573	0.892	6.763	0.264	0.576	1.132	0	2	1	1.396
49	4.71	0.631	0.984	6.763	0.291	0.601	1.138	0	2	1	1.429
50	4.30	0.622	0.972	6.763	0.287	0.422	0.012	0	2	0	0.300
51	4.76	0.569	0.895	6.763	0.265	0.409	0.012	0	2	0	0.277
52	3.65	0.576	0.907	6.763	0.268	0.500	0.015	0	2	0	0.283
53	4.68	0.575	0.907	6.763	0.268	0.408	0.012	0	2	0	0.280
54	4.69	0.585	0.921	6.763	0.272	0.464	0.014	0	2	0	0.286
55	4.91	0.734	1.143	6.763	2.072	0.526	1.121	1	2	1	3.193
56	4.98	0.646	0.993	6.763	0.294	0.498	0.015	0	2	0	0.309
57	4.93	0.641	0.985	6.763	0.291	0.494	0.015	0	2	0	0.306
58	4.13	0.742	1.141	6.763	0.506	0.448	0.013	0	3	0	0.520
59	4.18	0.639	0.992	6.763	0.293	0.428	0.013	0	2	0	0.306
60	4.86	0.572	0.897	6.763	0.265	0.402	0.012	0	2	0	0.277
61	5.64	0.549	0.856	6.763	0.127	0.429	0.013	0	1	0	0.139
62	5.63	0.545	0.848	6.763	0.125	0.485	0.014	0	1	0	0.140
63	5.84	0.754	1.156	6.763	2.095	0.458	0.014	1	2	0	2.109
64	6.33	0.719	1.103	6.763	2.045	0.465	0.014	1	2	0	2.059
65	4.97	0.739	1.132	6.763	2.073	0.573	1.131	1	2	1	3.205
66	5.27	0.740	1.132	6.763	0.502	0.464	0.014	0	3	0	0.516

67	5.52	0.691	1.063	6.763	0.314	0.458	0.014	0	2	0	0.328
68	4.76	0.691	1.063	6.763	0.314	0.384	0.011	0	2	0	0.326
69	4.75	0.665	1.023	6.763	0.303	0.479	0.014	0	2	0	0.317
70	5.75	0.724	1.115	6.763	0.495	0.465	0.014	0	3	0	0.508
71	6.22	0.727	1.117	6.763	0.496	0.494	0.015	0	3	0	0.510
72	5.78	0.713	1.097	6.763	0.487	0.481	0.014	0	3	0	0.501
73	5.29	0.713	1.103	6.763	0.326	0.585	1.134	0	2	1	1.460
74	4.94	1.247	2.088	6.763	5.729	0.528	0.016	2	4	0	5.745
75	5.20	0.725	1.122	6.763	0.498	0.502	0.015	0	3	0	0.512
76	7.40	0.726	1.185	7.290	0.488	0.483	0.013	0	3	0	0.501
77	6.17	0.767	1.181	6.763	0.524	0.440	0.013	0	3	0	0.537
78	5.74	0.867	1.336	6.625	2.069	0.618	1.142	1	1	1	3.211
79	4.57	0.610	0.910	6.625	0.137	0.349	0.011	0	1	0	0.148
80	4.92	1.003	1.662	6.625	2.756	0.474	0.014	1	3	0	2.770
81	5.47	0.937	1.375	6.625	6.435	0.583	1.134	3	3	1	7.570
82	4.84	0.652	1.013	6.625	0.306	0.355	0.011	0	2	0	0.317
83	4.55	0.643	0.954	6.625	0.288	0.345	0.010	0	2	0	0.298
84	4.71	0.668	0.992	6.625	0.299	0.427	0.013	0	2	0	0.312
85	5.18	1.030	1.562	6.625	4.768	0.692	1.159	2	3	1	5.927
86	4.59	0.675	0.989	6.625	1.974	0.521	1.120	1	2	1	3.093
87	4.12	0.596	0.884	6.625	0.000	0.527	0.016	0	0	0	0.016
88	4.36	0.615	0.915	6.625	0.138	0.563	1.130	0	1	1	1.268
89	4.28	0.602	0.902	6.625	0.136	0.339	0.010	0	1	0	0.146
90	5.10	0.593	0.879	6.625	0.133	0.355	0.011	0	1	0	0.143
91	4.58	0.552	0.816	6.625	0.123	0.354	0.011	0	1	0	0.134
92	7.50	0.655	1.068	7.171	0.149	0.430	0.012	0	1	0	0.161
93	4.82	0.572	0.849	6.625	0.128	0.479	0.014	0	1	0	0.143
94	5.74	0.517	0.764	6.625	0.000	0.383	0.012	0	0	0	0.012
95	4.55	0.580	0.878	6.625	0.133	0.368	0.011	0	1	0	0.144
96	5.14	0.706	1.060	6.625	0.320	0.486	1.112	0	2	1	1.432
97	7.40	0.695	1.107	7.171	0.309	0.443	0.012	0	2	0	0.321

98	6.20	0.787	1.207	6.625	2.151	0.523	1.120	1	2	1	3.272
99	5.71	0.620	0.915	6.625	0.276	0.531	0.016	0	2	0	0.292
100	5.79	0.984	1.501	6.625	2.890	0.486	0.015	1	4	0	2.905
101	4.66	0.630	0.932	6.625	0.281	0.429	0.013	0	2	0	0.294
102	4.40	0.709	1.108	6.625	0.669	0.510	0.015	0	4	0	0.685
103	4.47	0.618	0.938	6.625	0.425	0.431	0.013	0	3	0	0.438
104	4.50	0.552	0.823	6.625	0.000	0.378	0.011	0	0	0	0.011
105	4.51	0.522	0.775	6.625	0.000	0.426	0.013	0	0	0	0.013
106	4.77	0.681	1.048	6.625	0.158	0.421	0.013	0	1	0	0.171
107	4.44	0.589	0.876	6.625	0.264	0.444	0.013	0	2	0	0.278
108	4.61	0.703	1.067	6.625	0.483	0.385	0.012	0	3	0	0.495
109	4.68	0.615	0.917	6.625	0.277	0.392	0.012	0	2	0	0.289
110	4.65	0.613	0.918	6.625	0.277	0.399	0.012	0	2	0	0.289
111	4.96	0.550	0.818	6.625	0.000	0.397	0.012	0	0	0	0.012
112	5.23	0.859	1.300	6.625	2.447	0.572	1.132	1	3	1	3.579
113	4.41	0.662	1.001	6.625	0.302	0.408	0.012	0	2		0.315
114	4.23	0.730	1.111	6.625	0.335	0.394	0.012	0	2		0.347
115	5.01	0.635	0.951	6.625	0.144	0.535	0.016	0	1		0.160
116	7.30	0.919	1.485	7.171	2.747	0.829	1.189	1	4	1	3.936
117	7.22	0.837	1.344	7.171	2.587	0.754	2.323	1	4	2	4.909
118	5.41	1.175	1.847	6.625	5.465	0.691	1.159	2	4	1	6.624
119	5.63	0.670	0.980	6.625	1.818	0.775	1.178	1	1	1	2.996
120	6.69	0.641	1.014	7.171	0.283	0.684	1.156	0	2	1	1.439
121	5.07	0.610	0.905	6.625	0.137	0.463	0.014	0	1	0	0.151
122	6.24	0.743	1.113	6.625	2.079	0.621	1.143	1	2	1	3.222
123	5.91	0.710	1.077	6.625	2.035	0.580	1.134	1	2	1	3.169
124	5.55	0.611	0.916	6.625	0.277	0.593	1.137	0	2	1	1.413
125	4.74	0.638	0.963	6.625	0.145	0.592	1.136	0	1	1	1.282
126	4.34	0.567	0.834	6.625	0.126	0.556	1.128	0	1	1	1.254
127	5.77	0.578	0.872	6.625	0.132	0.565	1.130	0	1	1	1.262
128	4.18	0.683	1.028	6.625	0.310	0.411	0.012	0	2	0	0.323

129	7.70	0.616	0.964	7.171	0.269	0.606	1.138	0	2	1	1.407
130	5.35	0.568	0.868	6.625	0.000	0.442	0.013	0	0	0	0.013
131	5.38	0.776	1.220	6.625	2.328	0.421	0.013	1	3	0	2.341
132	5.04	0.619	0.913	6.625	0.276	0.493	0.015	0	2	0	0.291
133	4.91	0.637	0.949	6.625	0.143	0.440	0.013	0	1	0	0.156
134	4.36	0.768	1.159	6.625	0.525	0.411	0.012	0	3	0	0.537
135	4.92	0.700	1.041	6.625	0.314	0.395	0.012	0	2	0	0.326
136	4.32	0.598	0.924	6.625	0.000	0.447	0.014	0	0	0	0.014
137	5.37	0.536	0.809	6.625	0.000	0.412	0.012	0	0	0	0.012
138	4.82	0.695	1.056	6.625	0.319	0.373	0.011	0	2	0	0.330
139	4.87	0.650	0.948	6.625	0.286	0.374	0.011	0	2	0	0.298
140	4.99	0.540	0.822	6.625	0.124	0.422	0.013	0	1	0	0.137
141	5.22	0.632	0.973	6.625	0.294	0.523	0.016	0	2	0	0.309
142	4.67	0.654	1.005	6.625	0.455	0.451	0.014	0	3	0	0.469
143	4.80	0.644	0.957	6.625	0.289	0.343	0.010	0	2	0	0.299
144	4.86	0.695	1.071	6.625	0.647	0.478	0.014	0	4	0	0.661
145	5.02	0.704	1.092	6.625	0.495	0.364	0.011	0	3	0	0.506
146	4.80	0.875	1.384	6.625	2.502	0.415	0.013	1	3	0	2.514
147	4.84	0.666	1.005	6.625	0.304	0.431	0.013	0	2	0	0.317
148	4.40	0.616	0.910	6.625	0.275	0.700	1.161	0	2	1	1.436
149	4.44	0.704	1.034	6.625	0.468	0.467	0.014	0	3	0	0.482
150	5.26	0.634	0.922	6.625	0.278	0.587	1.135	0	2	1	1.414
151	4.73	1.266	1.937	6.625	5.116	0.584	0.018	2	2	0	5.134
152	5.02	0.661	0.954	6.625	1.949	0.599	1.138	1	2	1	3.086
153	5.24	0.644	0.928	6.625	1.925	0.777	2.334	1	2	2	4.259
154	6.71	0.617	0.982	7.171	0.274	0.410	0.011	0	2	0	0.285
155	7.36	0.483	0.761	7.171	0.000	0.407	0.011	0	0	0	0.011
156	7.21	0.746	1.182	7.171	2.405	0.611	0.017	1	4	0	2.422
157	7.50	0.495	0.771	7.171	0.107	0.373	0.010	0	1	0	0.118
158	5.40	0.648	0.977	6.625	0.295	0.392	0.012	0	2	0	0.307
159	4.79	0.622	0.941	6.625	0.142	0.539	0.016	0	1	0	0.158

160	5.18	0.660	1.007	6.625	0.304	0.440	0.013	0	2	0	0.317
161	5.32	0.798	1.240	6.625	2.359	0.555	0.017	1	3	0	2.376
162	5.26	0.647	1.086	6.625	0.328	0.531	0.016	0	2	0	0.344
163	5.50	0.474	0.772	6.625	0.000	0.422	0.013	0	0	0	0.013
164	5.68	0.609	0.948	6.625	0.143	0.395	0.012	0	1	0	0.155
165	5.29	1.025	1.646	6.625	3.018	0.599	0.018	1	4	0	3.036
166	5.43	0.545	0.822	6.625	0.124	0.418	0.013	0	1	0	0.137
167	5.93	0.505	0.780	6.625	0.118	0.507	0.015	0	1	0	0.133
168	5.84	0.608	0.941	6.625	0.284	0.535	1.123	0	2	1	1.407
169	5.27	0.559	0.837	6.625	0.000	0.406	0.012	0	0	0	0.012
170	5.56	0.602	0.913	6.625	0.000	0.542	0.016	0	0	0	0.016
171	6.03	0.599	0.899	6.625	0.136	0.393	0.012	0	1	0	0.148
172	5.17	0.568	0.868	6.625	0.000	0.357	0.011	0	0	0	0.011
173	5.17	0.718	1.108	6.625	1.885	0.388	0.012	1	1	0	1.897
174	5.14	0.546	0.823	6.625	0.000	0.484	0.015	0	0	0	0.015
175	4.57	0.505	0.825	6.625	0.249	0.212	0.006	0	2	0	0.255
176	4.79	0.733	1.125	6.625	0.510	0.482	0.015	0	3	0	0.524
177	5.03	0.774	1.204	6.625	0.727	0.551	0.017	0	4	0	0.743
178	4.30	0.601	0.900	6.625	0.136	0.473	0.014	0	1	0	0.150
179	4.46	0.699	1.080	6.625	0.163	0.293	0.009	0	1	0	0.172
180	6.14	0.617	0.924	6.625	0.140	0.618	1.142	0	1	1	1.282
181	5.19	0.782	1.188	6.625	0.359	0.324	0.010	0	2	0	0.368
182	4.51	0.413	0.742	7.405	0.000	0.429	0.012	0	0	0	0.012
183	5.16	0.609	1.084	7.405	0.439	0.446	0.012	0	3	0	0.451
184	4.73	0.595	1.073	7.405	0.435	0.456	0.012	0	3	0	0.447
185	5.14	0.568	1.020	7.405	0.275	0.438	0.012	0	2	0	0.287
186	5.23	0.601	1.084	7.405	0.439	0.454	0.012	0	3	0	0.451
187	6.96	0.460	0.936	8.004	0.117	0.460	0.011	0	1	0	0.128
188	5.16	0.550	0.987	7.405	0.267	0.426	0.012	0	2	0	0.278
189	5.08	0.563	1.014	7.405	0.274	0.470	0.013	0	2	0	0.287
190	5.01	0.560	1.011	7.405	0.273	0.459	0.012	0	2	0	0.286

191	5.49	0.357	0.658	7.405	0.000	0.215	0.006	0	0	0	0.006
192	5.16	0.563	1.021	7.405	0.276	0.657	1.149	0	2	1	1.425
193	7.52	0.545	1.043	8.004	0.391	0.454	0.011	0	3	0	0.402
194	6.02	0.444	0.834	7.405	0.113	0.215	0.006	0	1	0	0.118
195	6.44	0.357	0.659	7.405	0.000	0.479	0.013	0	0	0	0.013
196	6.92	0.401	0.821	8.004	0.103	0.232	0.006	0	1	0	0.108
197	6.77	0.400	0.825	8.004	0.103	0.215	0.005	0	1	0	0.108
198	4.72	0.621	1.058	7.405	1.906	0.406	0.011	1	2	0	1.917
199	4.78	0.638	1.090	7.405	1.932	0.427	0.012	1	2	0	1.944
200	4.68	0.661	1.135	7.405	1.967	0.469	0.013	1	2	0	1.980
201	4.88	0.665	1.146	7.405	1.974	0.465	0.013	1	2	0	1.987
202	4.11	0.657	1.126	7.405	0.456	0.452	0.012	0	3	0	0.468
203	4.83	0.652	1.107	7.405	2.101	0.471	0.013	1	3	0	2.113
204	4.83	0.634	1.068	7.405	2.066	0.465	0.013	1	3	0	2.079
205	4.83	0.637	1.080	7.405	2.075	0.476	0.013	1	3	0	2.088
206	4.36	0.630	1.075	7.405	0.435	0.492	0.013	0	3	0	0.449
207	4.80	0.652	1.118	7.405	1.954	0.506	0.014	1	2	0	1.967
208	4.77	0.606	1.026	7.405	1.883	0.452	0.012	1	2	0	1.895
209	4.93	0.657	1.131	7.405	1.963	0.441	0.012	1	2	0	1.975
210	4.50	0.655	1.122	7.405	2.110	0.423	0.011	1	3	0	2.121
211	4.43	0.633	1.081	7.405	1.925	0.447	0.012	1	2	0	1.937
212	4.55	0.686	1.182	7.405	2.165	0.475	0.013	1	3	0	2.178
213	4.57	0.563	0.951	7.405	0.257	0.571	0.015	0	2	0	0.272
214	4.55	0.671	1.186	7.405	2.151	0.519	0.014	1	3	0	2.166
215	4.96	0.561	0.945	7.405	0.255	0.504	0.014	0	2	0	0.269
216	4.58	0.507	0.845	7.405	0.228	0.496	0.013	0	2	0	0.242
217	4.43	0.546	0.918	7.405	0.248	0.459	0.012	0	2	0	0.260
218	4.88	0.526	0.875	7.405	0.236	0.513	1.116	0	2	1	1.353
219	4.08	0.546	0.911	7.405	0.246	0.522	0.014	0	2	0	0.260
220	4.56	0.597	1.014	7.405	0.411	0.516	1.117	0	3	1	1.528
221	4.52	0.497	0.828	7.405	0.224	0.521	0.014	0	2	0	0.238

222	5.20	0.505	0.841	7.405	0.114	0.389	0.011	0	1	0	0.124
223	4.61	0.534	0.894	7.405	0.242	0.617	1.140	0	2	1	1.382
224	4.66	0.509	0.845	7.405	0.228	0.446	0.012	0	2	0	0.240
225	4.40	0.507	0.843	7.405	0.228	0.591	1.134	0	2	1	1.362
226	4.63	0.531	0.879	7.405	0.237	0.501	0.014	0	2	0	0.251
227	4.45	0.503	0.838	7.405	0.226	0.408	0.011	0	2	0	0.237
228	6.94	0.758	1.428	8.386	2.268	0.439	0.010	1	3	0	2.279
229	4.97	0.661	1.192	7.715	0.309	0.484	0.013	0	2	0	0.321
230	5.41	0.707	1.294	7.715	2.043	0.311	0.008	1	2	0	2.051
231	5.32	0.650	1.157	7.715	0.300	0.478	0.012	0	2	0	0.312
232	5.48	0.704	1.287	7.715	2.037	0.587	1.133	1	2	1	3.170
233	5.59	0.611	1.085	7.715	0.281	0.567	0.015	0	2	0	0.296
234	5.46	0.623	1.114	7.715	0.289	0.424	0.011	0	2	0	0.300
235	5.40	0.502	0.866	7.715	0.000	0.519	0.013	0	0	0	0.013
236	5.31	0.517	0.892	7.715	0.116	0.508	1.115	0	1	1	1.230
237	4.65	0.574	0.987	7.715	0.256	0.743	1.168	0	2	1	1.424
238	4.68	0.547	0.927	7.715	0.360	0.644	1.145	0	3	1	1.506
239	5.65	0.498	0.854	7.715	0.000	0.546	0.014	0	0	0	0.014
240	4.72	0.591	0.969	7.715	0.251	0.474	0.012	0	2	0	0.264
241	5.59	0.560	0.969	7.715	0.251	0.567	0.015	0	2	0	0.266
242	4.93	0.643	1.156	7.715	0.300	0.538	0.014	0	2	0	0.314
243	5.66	0.597	1.016	7.715	0.263	0.616	1.139	0	2	1	1.402
244	5.24	0.679	1.137	7.715	2.121	0.516	0.013	1	3	0	2.135
245	4.98	0.579	0.970	7.715	0.251	0.526	0.014	0	2	0	0.265
246	4.41	0.605	1.027	7.715	0.399	0.534	0.014	0	3	0	0.413
247	4.26	0.638	1.104	7.715	0.429	0.533	0.014	0	3	0	0.443
248	4.27	0.651	1.124	7.715	0.437	0.533	0.014	0	3	0	0.451
249	4.90	0.640	1.137	7.715	0.590	0.908	1.205	0	4	1	1.795
250	5.22	0.636	1.146	7.715	0.149	0.514	0.013	0	1	0	0.162
251	5.38	0.636	1.092	7.715	0.425	0.733	1.166	0	3	1	1.590
252	5.43	0.581	1.004	7.715	0.260	0.583	0.015	0	2	0	0.275

253	5.79	0.582	0.990	7.715	0.385	0.661	1.149	0	3	1	1.534
254	5.15	0.551	0.963	7.715	0.250	0.532	0.014	0	2	0	0.263
255	5.62	0.637	1.108	7.715	0.431	0.555	0.014	0	3	0	0.445
256	5.49	0.439	0.766	7.715	0.099	0.398	0.010	0	1	0	0.110
257	4.88	0.464	0.825	7.715	0.107	0.421	0.011	0	1	0	0.118
258	4.43	0.415	0.723	7.715	0.000	0.432	0.011	0	0	0	0.011
259	4.64	0.418	0.713	7.715	0.000	0.410	0.011	0	0	0	0.011
260	4.77	0.462	0.804	7.715	0.104	0.503	0.013	0	1	0	0.117
261	4.50	0.446	0.765	7.715	0.000	0.444	0.012	0	0	0	0.012
262	5.22	0.454	0.772	7.715	0.000	0.446	0.012	0	0	0	0.012
263	4.55	0.418	0.698	7.715	0.000	0.496	1.112	0	0	1	1.112
264	4.71	0.418	0.698	7.715	0.000	0.535	1.121	0	0	1	1.121
265	5.67	0.456	0.817	7.715	0.106	0.579	1.131	0	1	1	1.237
266	5.14	0.476	0.809	7.715	0.000	0.619	1.140	0	0	1	1.140
267	5.06	0.481	0.829	7.715	0.107	0.636	1.144	0	1	1	1.251
268	4.65	0.460	0.789	7.715	0.102	0.469	0.012	0	1	0	0.114
269	4.88	0.455	0.783	7.715	0.101	0.463	0.012	0	1	0	0.113
270	4.62	0.484	0.837	7.715	0.000	0.514	1.116	0	0	1	1.116
271	5.65	0.550	0.993	7.715	0.129	0.594	1.134	0	1	1	1.263
272	5.00	0.450	0.762	7.715	0.000	0.561	0.015	0	0	0	0.015
273	4.88	0.514	0.856	7.715	0.111	0.415	0.011	0	1	0	0.122
274	4.60	0.472	0.835	7.715	0.108	0.454	0.012	0	1	0	0.120
275	4.48	0.433	0.761	7.715	0.099	0.512	1.116	0	1	1	1.214
276	5.36	0.489	0.844	7.715	0.109	0.406	0.011	0	1	0	0.120
277	5.04	0.540	0.931	7.715	0.121	0.475	0.012	0	1	0	0.133
278	5.43	0.519	0.918	7.715	0.119	0.514	1.116	0	1	1	1.235
279	5.17	0.627	1.164	7.715	1.778	0.579	1.131	1	1	1	2.908
280	5.10	0.554	1.009	7.715	0.131	0.372	0.010	0	1	0	0.140
281	6.22	0.582	1.065	7.715	0.138	0.416	0.011	0	1	0	0.149
282	5.29	0.576	1.045	7.715	0.271	0.488	0.013	0	2	0	0.284
283	5.08	0.637	1.153	7.715	0.299	0.617	0.016	0	2	0	0.315

284	4.51	0.581	1.009	7.715	0.261	0.432	0.011	0	2	0	0.273
285	5.83	0.538	0.952	7.715	0.123	0.503	0.013	0	1	0	0.136
286	5.73	0.518	0.905	7.715	0.117	0.477	0.012	0	1	0	0.130
287	5.70	0.557	0.931	7.715	0.362	0.597	1.135	0	3	1	1.497
288	6.03	0.581	0.980	7.715	0.381	0.571	1.129	0	3	1	1.510
289	5.95	0.567	0.954	7.715	0.371	0.583	1.132	0	3	1	1.503
290	4.64	0.489	0.799	7.715	0.000	0.447	0.012	0	0	0	0.012
291	4.80	0.495	0.813	7.715	0.000	0.444	0.012	0	0	0	0.012
292	4.39	0.511	0.832	7.715	0.216	0.421	0.011	0	2	0	0.227
293	4.39	0.510	0.831	7.715	0.215	0.460	0.012	0	2	0	0.227
294	3.82	0.428	0.726	7.715	0.000	0.413	0.011	0	0	0	0.011
295	4.17	0.424	0.717	7.715	0.000	0.458	0.012	0	0	0	0.012
296	4.66	0.459	0.788	7.715	0.000	0.419	0.011	0	0	0	0.011
297	4.74	0.469	0.799	7.715	0.000	0.429	0.011	0	0	0	0.011
298	4.69	0.454	0.765	7.715	0.000	0.416	0.011	0	0	0	0.011
299	5.03	0.581	1.057	7.715	0.137	0.438	0.011	0	1	0	0.148
300	6.68	0.660	1.274	8.386	0.456	0.499	0.012	0	3	0	0.468
301	6.92	0.679	1.328	8.386	2.154	0.582	1.130	1	3	1	3.285
302	5.97	0.582	0.994	7.715	0.386	0.431	0.011	0	3	0	0.398
303	7.25	0.627	1.156	8.386	0.276	0.400	0.010	0	2	0	0.285
304	5.94	0.577	0.988	7.715	0.384	0.606	1.137	0	3	1	1.521
305	5.61	0.581	0.993	7.715	0.386	0.524	0.014	0	3	0	0.400
306	6.66	0.600	1.117	8.386	0.400	0.576	1.129	0	3	1	1.528
307	5.55	0.607	1.028	7.715	0.400	0.521	0.014	0	3	0	0.413
308	6.57	0.646	1.220	8.386	0.436	0.392	0.009	0	3	0	0.446
309	5.46	0.698	1.207	7.715	0.469	0.229	0.006	0	3	0	0.475
310	5.48	0.734	1.288	7.715	0.501	0.252	0.007	0	3	0	0.507
311	5.39	0.663	1.162	7.715	0.452	0.440	0.011	0	3	0	0.463
312	8.31	0.609	1.110	8.386	0.397	0.486	0.012	0	3	0	0.409
313	5.85	0.742	1.329	7.715	0.517	0.678	1.153	0	3	1	1.670
314	7.76	0.598	1.065	8.386	1.852	0.482	0.011	1	2	0	1.864

315	7.90	0.600	1.068	8.386	1.855	0.425	0.010	1	2	0	1.865
316	8.15	0.605	1.081	8.386	1.863	0.408	0.010	1	2	0	1.872
317	5.36	0.546	0.976	7.715	0.126	0.514	0.013	0	1	0	0.140
318	5.33	0.559	0.993	7.715	0.129	0.540	0.014	0	1	0	0.143
319	5.24	0.539	0.959	7.715	0.124	0.591	1.134	0	1	1	1.258
320	6.95	0.560	0.995	8.386	1.797	0.824	1.184	1	2	1	2.981
321	6.87	0.563	1.001	8.386	1.802	0.492	0.012	1	2	0	1.814
322	6.79	0.556	0.980	8.386	1.789	0.501	0.012	1	2	0	1.801
323	6.68	0.581	1.056	8.386	1.832	0.664	1.149	1	2	1	2.981
324	8.34	0.570	1.088	8.386	0.130	0.632	1.141	0	1	1	1.271
325	8.46	0.568	1.079	8.386	0.129	0.594	1.133	0	1	1	1.262
326	8.29	0.581	1.110	8.386	0.132	0.634	1.142	0	1	1	1.274
327	8.29	0.593	1.080	8.386	0.258	0.629	1.141	0	2	1	1.398
328	7.93	0.590	1.073	8.386	0.256	0.611	1.137	0	2	1	1.393
329	8.16	0.624	1.143	8.386	0.409	0.654	1.146	0	3	1	1.555
330	7.63	0.559	1.051	8.386	0.125	0.640	1.143	0	1	1	1.269
331	8.06	0.637	1.170	8.386	1.916	0.671	1.150	1	2	1	3.066
332	4.48	0.563	1.012	7.978	0.254	0.393	0.010	0	2	0	0.264
333	4.39	0.533	0.934	7.715	0.242	0.376	0.010	0	2	0	0.252
334	4.30	0.618	1.093	7.715	0.283	0.423	0.011	0	2	0	0.294
335	4.19	0.658	1.179	7.715	0.306	0.517	0.013	0	2	0	0.319
336	4.41	0.599	1.055	7.715	0.274	0.512	1.116	0	2	1	1.389
337	4.46	0.549	0.946	7.715	0.245	0.463	1.105	0	2	1	1.350
338	4.55	0.545	0.937	7.715	0.243	0.479	1.108	0	2	1	1.351
339	4.43	0.533	0.936	7.715	0.243	0.392	0.010	0	2	0	0.253
340	4.57	0.496	0.835	7.715	0.108	0.357	0.009	0	1	0	0.117
341	4.91	0.493	0.806	7.715	0.104	0.602	1.136	0	1	1	1.240
342	4.76	0.493	0.807	7.715	0.105	0.626	1.141	0	1	1	1.246
343	4.59	0.563	0.968	7.715	0.251	0.399	0.010	0	2	0	0.261
344	4.41	0.588	1.026	7.715	0.266	0.372	0.010	0	2	0	0.275
345	4.43	0.454	0.741	7.715	0.000	0.471	0.012	0	0	0	0.012

346	4.63	0.594	1.078	7.715	0.140	0.662	1.150	0	1	1	1.289
347	4.94	0.443	0.757	7.715	0.000	0.459	0.012	0	0	0	0.012
348	5.83	0.601	1.004	7.715	0.260	0.607	1.137	0	2	1	1.397
349	4.90	0.495	0.811	7.715	0.105	0.388	0.010	0	1	0	0.115
350	4.72	0.487	0.795	7.715	0.103	0.378	0.010	0	1	0	0.113
351	4.63	0.651	1.088	7.715	1.933	0.402	0.010	1	2	0	1.943
352	4.95	0.648	1.080	7.715	1.928	0.429	0.011	1	2	0	1.939
353	4.56	0.536	0.888	7.715	0.230	0.341	0.009	0	2	0	0.239
354	4.44	0.559	0.934	7.715	0.363	0.338	0.009	0	3	0	0.372
355	8.35	0.735	1.366	8.386	2.224	0.515	0.012	1	3	0	2.237
356	7.40	0.783	1.605	8.386	2.357	0.547	1.122	1	3	1	3.479
357	5.80	0.613	1.072	7.715	0.417	0.630	1.142	0	3	1	1.559
358	7.30	0.611	1.140	8.386	0.408	0.452	0.011	0	3	0	0.419
359	7.68	0.599	1.121	8.386	0.401	0.537	1.120	0	3	1	1.521
360	5.79	0.767	1.424	7.715	0.738	0.434	0.011	0	4	0	0.750
361	5.59	0.710	1.287	7.715	0.667	0.464	0.012	0	4	0	0.679
362	5.40	0.579	0.974	7.715	0.379	0.535	0.014	0	3	0	0.393
363	4.36	0.594	1.001	7.715	0.389	0.539	0.014	0	3	0	0.403
364	4.66	0.554	0.941	7.715	0.122	0.486	0.013	0	1	0	0.135
365	5.00	0.513	0.844	7.715	0.219	0.505	0.013	0	2	0	0.232
366	4.60	0.507	0.847	7.715	0.110	0.436	0.011	0	1	0	0.121
367	4.71	0.624	1.085	7.715	0.281	0.588	1.133	0	2	1	1.414
368	4.61	0.720	1.340	7.715	0.521	0.460	0.012	0	3	0	0.533
369	4.68	0.612	1.040	7.715	0.404	0.483	0.013	0	3	0	0.417
370	4.84	0.717	1.323	7.715	0.686	0.513	0.013	0	4	0	0.699
371	4.22	0.653	1.186	7.715	0.308	0.449	0.012	0	2	0	0.319
372	7.43	0.548	0.991	8.386	0.236	0.507	0.012	0	2	0	0.248
373	7.32	0.534	0.950	8.386	0.340	0.449	0.011	0	3	0	0.350
374	7.43	0.530	0.943	8.386	0.225	0.888	1.199	0	2	1	1.424
375	7.36	0.551	1.014	8.386	0.242	0.751	1.168	0	2	1	1.410
376	4.37	0.597	1.070	7.715	0.277	0.474	0.012	0	2	0	0.290

377	5.97	0.648	1.165	7.715	0.302	0.656	1.148	0	2	1	1.450
378	6.59	0.629	1.237	8.386	0.443	0.678	1.152	0	3	1	1.594
379	4.99	0.557	0.983	7.715	0.255	0.514	0.013	0	2	0	0.268
380	5.63	0.636	1.067	7.715	0.415	0.445	0.012	0	3	0	0.426
381	5.46	0.624	1.032	7.715	0.268	0.472	0.012	0	2	0	0.280
382	4.88	0.609	1.002	7.715	0.260	0.518	1.117	0	2	1	1.377
383	5.76	0.634	1.059	7.715	0.412	0.609	1.138	0	3	1	1.549
384	4.47	0.562	0.923	7.715	0.239	0.581	1.131	0	2	1	1.370
385	4.63	0.625	1.088	7.715	0.423	0.349	0.009	0	3	0	0.432
386	4.78	0.661	1.144	7.715	0.445	0.508	0.013	0	3	0	0.458
387	4.92	0.529	0.898	7.715	0.233	0.597	1.135	0	2	1	1.368
388	5.04	0.554	1.009	7.715	0.131	0.540	1.122	0	1	1	1.253
389	4.91	0.562	1.026	7.715	0.133	0.788	1.178	0	1	1	1.311
390	4.91	0.571	1.041	7.715	0.135	0.477	0.012	0	1	0	0.147
391	5.36	0.719	1.360	7.715	2.072	0.683	1.154	1	2	1	3.226
392	4.79	0.907	1.618	7.715	4.444	0.649	1.147	2	3	1	5.590
393	5.12	0.780	1.281	7.715	2.112	0.606	1.137	1	2	1	3.249
394	5.25	0.494	0.830	7.715	0.108	0.502	0.013	0	1	0	0.121
395	6.13	0.672	1.165	7.715	0.453	0.580	1.131	0	3	1	1.584
396	6.55	0.698	1.309	8.123	0.483	0.563	1.126	0	3	1	1.610
397	4.26	0.645	1.098	7.715	1.929	0.390	0.010	1	2	0	1.940
398	4.21	0.553	0.943	7.715	0.245	0.524	1.118	0	2	1	1.363
399	4.81	0.697	1.186	7.715	0.461	0.485	0.013	0	3	0	0.474
400	4.90	0.662	1.160	7.715	0.451	0.404	0.010	0	3	0	0.462
401	4.81	0.634	1.092	7.715	0.425	0.452	0.012	0	3	0	0.436
402	4.85	0.713	1.221	7.715	0.475	0.782	1.177	0	3	1	1.652
403	6.20	0.961	1.647	7.715	6.522	0.540	0.014	3	3	0	6.536
404	5.13	0.476	0.794	7.715	0.000	0.596	1.135	0	0	1	1.135
405	4.94	0.901	1.701	7.715	2.563	0.435	0.011	1	3	0	2.574
406	4.73	0.646	1.141	7.715	0.444	0.508	0.013	0	3	0	0.457
407	6.31	0.580	1.059	7.715	0.137	0.672	1.152	0	1	1	1.289

408	6.07	1.185	2.053	6.611	5.613	0.774	1.178	2	4	1	6.791
409	6.68	1.208	2.657	8.386	7.891	0.831	1.186	3	4	1	9.077
410	7.04	1.309	2.779	8.386	5.944	0.965	1.216	2	4	1	7.160
411	5.18	0.722	1.281	7.715	0.498	0.454	0.012	0	3	0	0.510
412	4.72	0.736	1.303	7.715	0.507	0.595	0.015	0	3	0	0.522
413	5.00	0.636	1.066	7.715	0.276	0.362	0.009	0	2	0	0.286
414	4.54	0.635	1.057	7.715	0.274	0.362	0.009	0	2	0	0.283
415	5.03	0.673	1.166	7.715	0.453	0.502	0.013	0	3	0	0.466
416	4.60	0.758	1.371	7.715	2.291	0.344	0.009	1	3	0	2.300
417	5.88	0.827	1.579	7.715	2.441	0.575	0.015	1	3	0	2.456
418	7.01	1.571	3.669	8.386	3.446	0.562	0.013	1	2	0	3.459
419	4.71	0.599	1.027	7.715	0.266	0.726	1.164	0	2	1	1.430
420	5.22	0.531	0.922	7.715	0.119	0.559	1.126	0	1	1	1.246
421	4.69	0.775	1.316	7.715	2.457	0.603	1.136	1	4	1	3.593
